# Investigating the potential role of TRPA1 in locomotion and cardiovascular control during hypertension

**DOI:** 10.1002/prp2.52

**Published:** 2014-06-23

**Authors:** Jennifer V Bodkin, Pratish Thakore, Aisah A Aubdool, Lihuan Liang, Elizabeth S Fernandes, Manasi Nandi, Domenico Spina, James E Clark, Philip I Aaronson, Michael J Shattock, Susan D Brain

**Affiliations:** 1 Cardiovascular Division, BHF Centre of Excellence and Centre of Integrative Biomedicine, School of Medicine, King’s College London London, SE1 9NH, U.K; 2 Pharmaceutical Sciences Division, School of Biomedical Sciences, King’s College London London, SE1 9NH, U.K; 3 Programa de Pós-Graduação em Biologia Parasitária, Universidade Ceuma São Luís, Brazil; 4 Asthma, Allergy and Lung Biology Division, School of Medicine, King’s College London London, SE1 1UL, U.K

**Keywords:** Blood pressure, cinnamaldehyde, hypertension, locomotion, TRPA1

## Abstract

Radiotelemetry was used to investigate the in vivo cardiovascular and activity phenotype of both TRPA1 (transient receptor potential ankyrin 1) wild-type (WT) and TRPA1 knockout (KO) mice. After baseline recording, experimental hypertension was induced using angiotensin II infusion (1.1 mg^−1^ kg^−1^ a day, for 14 days). TRPA1 WT and KO mice showed similar morphological and functional cardiovascular parameters, including similar basal blood pressure (BP), heart rate, size, and function. Similar hypertension was also displayed in response to angiotensin II (156 ± 7 and 165 ± 11 mmHg, systolic BP ± SEM, *n* = 5–6). TRPA1 KO mice showed increased hypertensive hypertrophy (heart weight:tibia length: 7.3 ± 1.6 mg mm^−1^ vs. 8.8 ± 1.7 mg mm^−1^) and presented with blunted interleukin 6 (IL-6) production compared with hypertensive WT mice (151 ± 24 vs. 89 ± 16 pg mL^−1^). TRPA1 expression in dorsal root ganglion (DRG) neurones was upregulated during hypertension (163% of baseline expression). Investigations utilizing the TRPA1 agonist cinnamaldehyde (CA) on mesenteric arterioles isolated from näive mice suggested a lack of TRPA1-dependent vasoreactivity in this vascular bed; a site with notable ability to alter total peripheral resistance. However, mesenteric arterioles isolated from TRPA1 KO hypertensive mice displayed significantly reduced ability to relax in response to nitric oxide (NO) (*P* < 0.05). Unexpectedly, naïve TRPA1 KO mice also displayed physical hyperactivity traits at baseline, which was exacerbated during hypertension. In conclusion, our study provides a novel cardiovascular characterization of TRPA1 KO mice in a model of hypertension. Results suggest that TRPA1 has a limited role in global cardiovascular control, but we demonstrate an unexpected capacity for TRPA1 to regulate physical activity.

## Introduction

The transient receptor potential ankyrin 1 (TRPA1) receptor is activated by a range of potentially injurious exogenous and endogenous compounds, leading to the release of neuropeptides (Bodkin and Brain 2010; Earley 2012). Since its identification it has developed a significant reputation as a mediator of pain and inflammation (Jaquemar et al. 1999). TRPA1 is classically expressed on C-fiber sensory neurones (Story et al. 2003), but expression in other locations has been reported, including rat cerebral artery endothelial cells (Earley et al. 2009). In line with the role for TRPA1 in inflammation, several groups demonstrate TRPA1 agonists to cause vasorelaxation or vasodilation, occurring in a range of species and vascular beds (Bodkin and Brain 2010). TRPA1-induced vasorelaxation is commonly identified to be neuropeptide mediated (Louis et al. 1989; Morris et al. 1999; Hikiji et al. 2000; Grant et al. 2005; Namer et al. 2005; Graepel et al. 2011; Kunkler et al. 2011), while other studies propose direct changes in calcium handling (Yanaga et al. 2006; Earley et al. 2009; Xue et al. 2011).

Cinnamaldehyde (CA) is a commonly used TRPA1 agonist, selected due to its relative selectivity and potency (Bandell et al. 2004). It has been shown to cause vasorelaxation and vasodilation in several studies (VanderEnde and Morrow 2001; Namer et al. 2005; Yanaga et al. 2006; Pozsgai et al. 2010; Xue et al. 2011). Our group has also recently demonstrated CA induced ex vivo vasorelaxation and in vivo vasodilation responses to be significantly reduced in TRPA1 knockout (KO) mice (Pozsgai et al. 2010). Moreover, intravenous administration of CA initiated an autonomic vaso-vagal response which significantly reduced in TRPA1 KO mice (Pozsgai et al. 2010). Similar blood pressure (BP) reflexes have previously been observed in other species following systemic administration of CA (Harada et al. 1975, 1982), alongside changes in autonomic drive and heart rate (Iwasaki et al. 2008; Hazari et al. 2011).

Our group has recently demonstrated the protective role of the neuropeptide calcitonin gene-related peptide (CGRP) in hypertension (Smillie et al. 2014), so we hypothesized that TRPA1 KO mice may show susceptibility to angiotensin II-induced hypertension and related morbidities. Basal and hypertensive cardiovascular characteristics were generally similar in TRPA1 (wild-type) WT and KO mice. However, some significant differences were seen in TRPA1 KO mice during hypertension; notably, a trend for increased hypertrophy and a blunted increase in IL-6. Little difference in the pharmacology of mesenteric arterioles from either genotype was seen. Our most surprising and striking finding was that TRPA1 KO mice demonstrated a previously undescribed hyperactivity trait.

## Materials and Methods

### Animals

Experiments were carried out according to the Animals (Scientific Procedures) Act 1986 and were ethically approved by the local Research Ethics Committee. Animals were bred and housed in a climatically controlled environment, on a 12-h light (7 am–7 pm)/dark cycle, with free access to a normal diet and water. Initial pairs of mixed genetic background TRPA1 WT and KO mice (C57BL/6B6129P1/F2J) were as described in Pozsgai et al. (2010). Both sexes of mice were used in pairs, aged between 2 and 3 months, and weighing more than 20 g. Mice were age matched and also littermates where possible. Appropriate handling and care was taken throughout, with postsurgical analgesia provided. The number of animals used per experiment is stated in each figure.

### Wire myography

First-order mesenteric artery branches (∼150–250 *μ*m diameter) were isolated from CD1, TRPA1 WT, or TRPA1 KO mice in ice-cold-modified Krebs’-Henseleit solution (118 mmol/L NaCl, 24 mmol/L NaHCO_3_, 1 mmol/L MgSO_4_, 4 mmol/L KCl, 0.5 mmol/L NaH_2_PO_4_, 5.5 mmol/L glucose, and 2.5 mmol/L CaCl_2_, all salts from Sigma-Aldrich, Gillingham, U.K.). They were cleansed of fatty tissue, mounted, and normalized to normal peripheral artery tension (13.3 kPa) on a wire myograph (DMT 610M or 620) using 0.025-mm stainless steel wires, as in Pozsgai et al. (2010). Vessels were maintained throughout the experiment in 37°C Krebs gassed with air/5% CO_2_. Vessel viability was assessed by determining constriction to 80 mmol/L K^+^ Krebs’ solution (reduction of NaCl to 38 mmol/L, all other constituents remain unchanged), and endothelial function was assessed using carbachol (10 *μ*mol/L, in double deionized water, Sigma-Aldrich), where a relaxation of >60% was used as a positive threshold. Cumulative concentration-response curves were constructed to the *α*_1_-adrenergic receptor agonist phenylephrine (30 nmol/L to 100 *μ*mol/L in double deionized water, Sigma-Aldrich) and the thromboxane A_2_ (TxA_2_) analogue, U46619 (100 pmol/L to 300 nmol/L, ethanol in double deionized water; Enzo Life Sciences, Exeter, U.K.). Data are expressed as percentage of 80 mmol/L K^+^ Krebs’-induced contraction. For relaxation studies, tissues were submaximally preconstricted with U46619 (10 nmol/L), then exposed to cumulative additions of either CA (trans-cinnamaldehyde >95%, Sigma-Aldrich, 3–1000 *μ*mol/L, ethanol), human *α*CGRP (1–300 nmol/L in 0.01% bovine serum albumin [BSA], double deionized water, Bachem, Bubendorf, Switzerland), carbachol (100 pmol/L to 30 *μ*mol/L in double deionized water, Sigma-Aldrich), or sodium nitroprusside ([SNP],100 pmol/L to 30 *μ*mol/L in double deionized water, Sigma-Aldrich). Data are expressed as percentage relaxation of preconstricted tone. In some experiments, additional pharmacological tools were used, this included the CGRP receptor antagonist CGRP_8-37_ (3 *μ*mol/L in 0.01% BSA in double deionized water, 5 min before preconstriction; Bachem) and the TRPA1 antagonist HC030031 (50 *μ*mol/L in dimethyl sulfoxide [DMSO] and Krebs), 15 min before preconstriction; Tocris Bioscience, Bristol, U.K.). Results from multiple vessels per mouse were averaged to produce each N. A four parameter logistic equation enabled us to plot response curves, where *E*_Max_ and *pEC*_50_ values were extrapolated and analyzed to determine statistical significance (GraphPad Prism 5, GraphPad Software Inc., La Jolla, CA).

### Radiotelemetry

BP, heart rate, and spontaneous activity counts were measured using a telemetry device (PA-C10, DSI, NL), with the catheter tip placed in the left carotid artery and advanced toward the aortic arch. The transmitter body was placed subcutaneously in the right flank, similar to Marshall et al. (2013). Implantation surgery was conducted using aseptic techniques under a surgical level of isoflurane anesthesia (Abbott Laboratories, Maidenhead, U.K.). Pain relief was administered before the procedure in the form of intramuscular buprenorphine (50 *μ*g kg^−1^, Vetergesic). Baseline data were collected at days 10–13 post surgery using DSI software (DSI Dataquest A.R.T., Data Sciences International, s’Hertogenbosch, the Netherlands).

### Experimental hypertension

On day 14 post telemetry surgery, induction of experimental hypertension was initiated with 14-day continuous infusion of angiotensin II (1.1 mg^−1^ kg^−1^ per day, Sigma-Aldrich) by subcutaneously implanted osmotic minipump (1002; Alzet, Cupertino, CA), with control mice also receiving a pump, as in Smillie et al. (2014).

Telemetered animals were monitored for hypertension progression immediately following implantation for 14 days. Mice were then terminated, 100-*μ*L aliquots of plasma were snap frozen, and stored at −80°C. DRG, heart, mesenteric arteries, lower aorta portion, and brain segments were individually stored in RNA *later* (Sigma-Aldrich), as per manufacturer’s instructions. Mesenteric arterioles were also collected and examined for pharmacological changes using myography.

### Voluntary wheel running

Mixed genotype littermate mice (2 months of age) had plastic wheels (13.4 cm diameter) added to their home cage for acclimatization. After 3 weeks, mice pairs were separated by genotype and moved to a quiet room. Daily running (km) and speed of wheel turning (km h^−1^) were recorded using a rotational counter for a period of 7 days/6 nights. No significant differences in activity between male and female mice were observed.

### Echocardiography

Ventricular mass, wall thicknesses, and functional measurements were collected from 3-month-old mice, either naïve or telemetered animals (both preimplantation of osmotic minipumps and at the end of 14-day angiotensin II infusion). Cardiac echocardiography was performed using a VEVO 770 with a mouse scan head (RMV 707B, 30 Hz; VisualSonics, Inc., Toronto, ON, Canada), carried out as in Respress and Wehrens (2010). The examination lasted less than 20 min. M-mode and B-mode images were analyzed using the leading edge to leading edge method over five sinus beats. Ventricle mass and thicknesses are expressed as ratio to mouse body mass, whereas functional measures are expressed in their respective units.

### Plasma cytokine quantification

Interleukin 1 (IL-1), tumor necrosis factor alpha (TNF*α*), Interleukin 12 (IL-12), murine Interleukin 8 (KC), interferon gamma (IFN*γ*), Interleukin 10 (IL-10), and IL-6 plasma protein levels were measured (Russell et al. 2009), using a seven spot multiplex mouse pro-inflammatory assay kit (Meso-Scale Discovery, Gaithersburg, MD).

### Real-time reverse transcription polymerase chain reaction

Real-time reverse transcription polymerase chain reaction (RT-qPCR) was carried out as in Smillie et al. (2014). Total ribonucleic acid (RNA) was extracted from tissue using the Qiagen RNeasy Microarray tissue mini Kit (Qiagen, Manchester, U.K.) and Qiagen’s TissueLyser LT. A Reverse transcription enzyme kit with RNAase inhibitor (Life Technologies, Paisley, U.K.) and thermal cycler (Life Technologies) was used for reverse transcription to complementary DNA (cDNA). Quantitative PCR (qPCR) was then conducted using a SYBR-green-based PCR mix (Sensi-Mix, SYBR-green No ROX; Bioline Reagents Ltd., London, U.K.) pipetted into 100-well gene disks (Qiagen) by an automated robot (CAS1200; Qiagen Corbett, Manchester, U.K.), followed by PCR in a Corbett Rotorgene 6000. Settings were as follows; hold: 10 min at 95°C; cycling: 45 cycles: 10 sec at 95°C, 15 sec at 57°C, and 5 sec at 72°C; melt: 68–90°C. Data were collected as copies/*μ*L and normalized against murine phospholipase A2 (PLA_2_G12A), actin, and glyceraldehyde-3-phosphate dehydrogenase (GAPDH) expression using GeNorm3.4 software (Vandesompele et al. 2002). The following primers were from Sigma-Aldrich (5′–3′): *TRPA1*: F-AGGTGATTTTTAAAACATTGCTGAG, R-CTCGATAATTGATGTCTCCTAGCAT; *IL-6*: F-GCTACCAAACTGGATATAATCAGGA, R- CCAGGTAGCTATGGTACTCCAGAA; *PLA*_*2*_*G12A*: F-TGGATATAAACCATCTACCA, R-GGGAAGGGATACCTATGTTCAGA; *Actin*: F-CACAGCTTCTTTGCAGCTCCTT, R-TCAGGATACCTCTCTTGCTCT; *GAPDH*: F- GGTCATCCCAGAGCTGAACG, R- TTGCTGTTGAAGTCGCAGGA.

### Data analysis

Data are shown as mean ± standard error of the mean (SEM). Data are presented and tested for significance using GraphPad Prism 5. Multiple group analysis was performed by one- or two-way analysis of variance (ANOVA) with Sidak’s or Bonferroni’s post hoc test, or a two-tailed unpaired *t*-test for two groups. For nonparametric datasets, a Mann–Whitney *U*-test was used. A one-tailed unpaired *t*-test was used to compare TRPA1 WT and KO spontaneous activity levels in the final week of angiotensin II infusion. Statistical tests and outcomes are described within each figure legend.

## Results

### Naïve TRPA1 WT and KO mice display indistinguishable cardiovascular phenotypes

**Figure 1:**
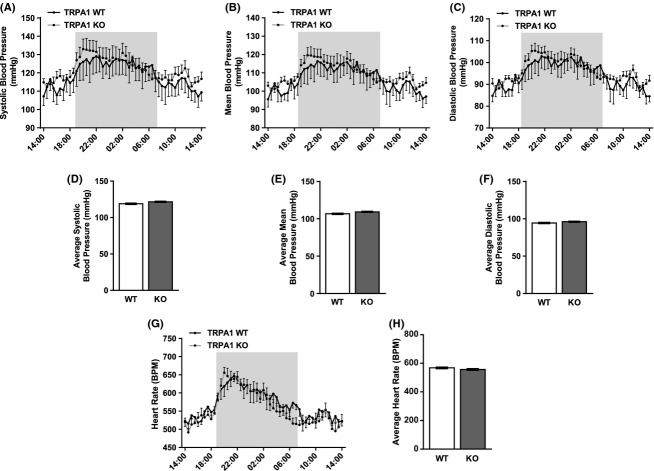
Basal hemodynamics in TRPA1 WT and KO mice measured by telemetry. Systolic, mean, and diastolic pressure (A–C) with heart rate (G) readings were collected 10–13 days after implantation of telemetry probe PA-C10 with an aortic arch placed catheter. Mice experience a 12/12 h light/dark cycle, with the dark cycle shown in the shaded area. Data are shown as hourly averages collected from three daily cycles. (D–H) show respective datasets shown as 24-h averages. *N* = 7–10 mice for all, data shown ± SEM. Groups are not significantly different when tested with appropriate statistical analysis.

We previously reported similar baseline BP and heart rate values in anesthetized TRPA1 WT and KO mice (Pozsgai et al. 2010). We now confirm these findings using radiotelemetry from conscious, unrestrained TRPA1 WT and KO mice (Fig. 1). Averaged from three consecutive days and plotted half hourly, TRPA1 KO mice showed similar systolic, mean, and diastolic BP profiles (Fig. 1A–C). These similarities were also reflected in the 24-h averages shown in panels D–F. The heart rate profile and 24-h average were also similar between TRPA1 WT and KO mice (Fig. 1G–H). Clear diurnal rhythms were seen within the data, showing peaks in BP and heart rate shortly after switch-over to darkness; a pattern expected from nocturnal animals.

Telemetered and naïve mice were assessed for cardiac morphology and function in vivo using echocardiography. Table 1 comprises some key measures, namely body weight (BW), left ventricle (LV) mass, cardiac output (CO), stroke volume (SV), ejection fraction (EF), and fractional shortening (FS); which were found to be almost identical in TRPA1 WT and KO mice. Ventricular wall thicknesses were also assessed, and were indistinguishable (WT data are shown in Fig. 4C). These findings were confirmed by measuring heart mass at necropsy, where ratios to BW and tibia length showed heart mass to be similar in WT and KO mice (Table 1).

**Table 1:** Basal body weight, heart size, and function in TRPA1 WT and KO mice

	TRPA1 WT	TRPA1 KO
Body weight	24.34 ± 1.00	25.17 ± 0.93
(g)	*N* = 27	*N* = 27
HW:BW	4.69 ± 0.18	4.87 ± 0.20
(mg)	*N* = 11	*N* = 13
HW:Tibia length	67.79 ± 6.21	73.02 ± 4.71
(*μ*g mm^−1^)	*N* = 8	*N* = 9
LV:BW	4.23 ± 0.22	4.32 ± 0.24
(mg g^−1^)	*N* = 27	*N* = 29
(echocardiography)		
Cardiac output	17.27 ± 0.93	17.14 ± 0.93
(mL min^−1^)	*N* = 27	*N* = 29
Stroke volume	34.44 ± 1.68	35.20 ± 1.54
(*μ*L)	*N* = 27	*N* = 29
Ejection fraction	66.73 ± 1.76	64.59 ± 1.57
(%)	*N* = 27	*N* = 29
Fractional shortening	35.67 ± 1.36	34.08 ± 1.07
(%)	*N* = 27	*N* = 29

Total body mass (BW), heart mass (HW), and tibia length were measured in 3-month-old mice at necropsy, while LV mass-to-body weight ratio (LV:BW) and all functional measures were previously calculated using in vivo echocardiography. Units and N as indicated ± SEM.

### Naïve TRPA1 KO mice display an unexpectedly increased preference for activity when compared with TRPA1 WT littermates

**Figure 2:**
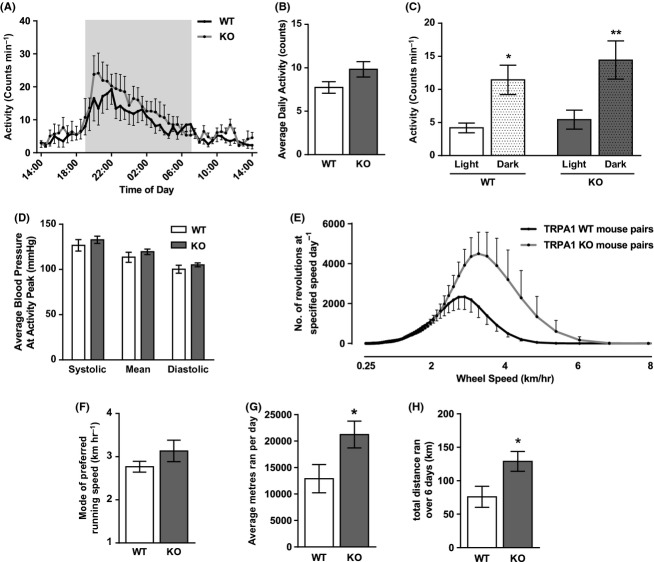
Basal spontaneous activity counts in TRPA1 WT and KO mice measured by telemetry and during voluntary wheel running. Activity counts were collected by telemetry on days 10–13 following implantation of a PA-C10 probe and presented as half hourly (A) and 24-hourly (B) averages, and also as averages during the 12-h light and dark phases (C). Corresponding BP data were collected from a catheter in the aortic arch and average pressures measured during the peak of activity (8–8.30 pm) are presented in (D). *N* = 7–10 mice. In a separate set of experiments, naïve mice housed in pairs were allowed access to an activity wheel. After a 2-week acclimatization period, mice were moved to a secluded room and data on wheel usage were collected over a 6-night/7-day period. (E) shows a histogram of wheel turning speed and (F) shows the mode of these data, describing the preferred running speed of the littermate mouse pairs. G and H show the average distance ran per day and the total distance ran over the experimental period. *N* = 6 WT pairs, 4 KO pairs. All data shown ± SEM. Statistics in C are from one-way ANOVA with Sidak’s post hoc test, *P < 0.05 or **P < 0.01 compared to respective light-time count. Statistics G and H are from a nonparametric, one-tailed Mann–Whitney *U-*test. *P < 0.05.

The PA-C10 telemetry probe also measures spontaneous activity counts, collected in parallel with BP measurements. Figure 2A shows the profile of activity counts, averaged over 3 days of recording, and plotted every half an hour. Figure 2B shows the average number of counts over 24 h. TRPA1 KO mice participated in increased spontaneous activity compared to TRPA1 WT mice. Data was split to show activity counts collected during the light and dark phases (Fig. 2C), where the majority of activity occurred during the nighttime. This showed that TRPA1 KO mice did not participate in more prolonged activity (i.e., disturbed sleep), but moved about more intensely during waking hours. In order to address the potential effect of activity on cardiovascular parameters, average blood pressures collected during the peak of activity (8–8.30 pm) were plotted (Fig. 2D). TRPA1 WT and KO mice showed no significant differences in BP despite clear differences in activity levels.

To investigate this further, pairs of naïve mice were allowed free access to a running wheel in their cage. After 3 weeks of acclimatization, mice were moved to a secluded room and their running behavior measured remotely. Figure 2E shows a histogram of wheel turning speeds and the mode of preferred running speed, derived from this, is shown in Figure 2F. Consistent with indications from telemetered animals, TRPA1 KO mice showed a preference for running at faster speeds than their WT littermates, therefore conducted more intense periods of voluntary activity. This is reflected in the average daily distance and the total weekly distance ran by mouse pairs (Fig. 2G–H). Therefore, our findings show that when given the opportunity to exercise, TRPA1 KO mice preferred to run further, and faster than TRPA1 WT mice.

### TRPA1 WT and KO mice show similar hypertension following angiotensin II infusion, while activity levels are further exacerbated in TRPA1 KO mice

**Figure 3:**
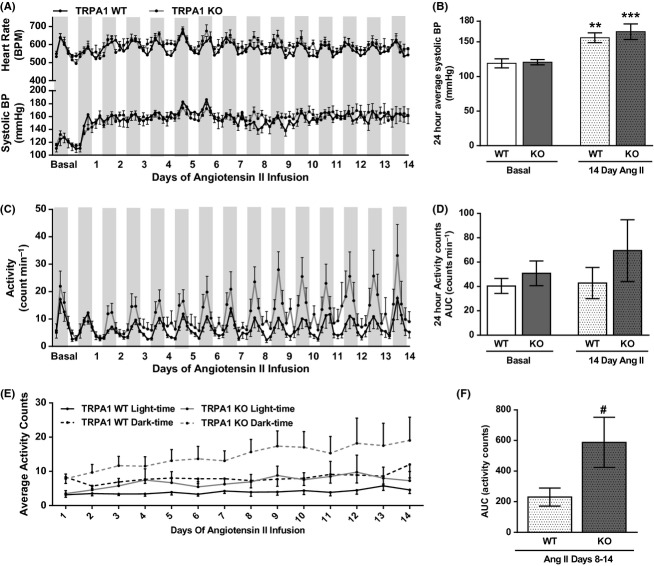
Hemodynamic and locomotion characteristics in TRPA1 WT and KO mice during 14 days of angiotensin II infusion, measured by telemetry. Angiotensin II was infused at 1.1 mg kg^−1^ a day from a subcutaneously implanted osmotic minipump. BP and activity counts were collected by telemetry, with basal data averaged from the 3 days immediately before implantation of the minipump. Dark stripes indicate nocturnal period. A shows systolic BP and heart rate measurements collected over 14 days of angiotensin II infusion and plotted as 4-h averages. 24h averages of basal data and day 14 of angiotensin II infusion are shown in B. Similarly, presented measurements of activity counts are shown in C and D. This profile is further split to show average activity counts during the light and dark phases in (E). As TRPA1 KO activity shows increases during angiotensin II infusion, (F) shows the average activity of TRPA1 WT and KO mice during the second week of angiotensin II infusion. *N* = 7–10 mice, all data shown ± SEM. Statistics in B and D are from a two-way ANOVA, with Sidak’s post hoc test. **P < 0.01. *** *P* < 0.001 compared to respective baseline. In F, TRPA1 WT and KO activity levels in days 8–14 of angiotensin II infusion are compared with a one-tailed, unpaired *t*-test, # *P* < 0.05.

TRPA1 KO mice were infused with a pressor dose of angiotensin II for 14 days (1.1 mg^−1^ kg^−1^ day), similar to that used in Smillie et al. (2014). Figure 3A shows the profile of hypertension onset, in terms of systolic BP and heart rate, plotted as 4-h averages. Basal data are also shown for comparison. Hypertension was established within the first few days of angiotensin II infusion, with onset and magnitude being similar in TRPA1 WT and KO mice. No changes in heart rate were observed over this period (Fig. 3A). Average 24-h systolic BP was significantly elevated after 14 days of angiotensin II infusion in TRPA1 WT and KO mice, but with similar magnitudes (Fig. 3B).

Despite similar BP responses in TRPA1 WT and KO mice, the differences in spontaneous activity levels were exacerbated during angiotensin II infusion. Spontaneous activity counts were concomitantly measured with BP and are shown in Figure 3C. After an initial reduction in activity following surgery, TRPA1 KO mice reaffirmed trends for increased activity compared to WT mice. Furthermore, as the infusion period progressed, TRPA1 WT mice showed little change in activity level, whereas TRPA1 KO mice showed an ever-increasing amount of activity. This data is summarized in Figure 3D and F, consistently showing trends for increased activity levels in TRPA1 KO mice. The magnitude of these differences was increased during the final infusion day (Fig. 3D) and spontaneous activity levels during the second week of angiotensin II infusion were significantly higher in TRPA1 KO mice compared to WT mice (Fig. 3F). When 14-day activity profiles were separated to show activity counts occurring in the light and dark phases, TRPA1 KO mice displayed higher levels of nocturnal activity (Fig. 3E). In keeping with previous data, TRPA1 KO mice showed more intense nocturnal activity than WT mice, which was further enhanced during angiotensin II infusion.

### TRPA1 KO mice show a trend for increased hypertrophy and significant blunting in the production of IL-6 despite a broadly similar hypertensive response

TRPA1 activation has strong links to inflammation. We have recently shown genetic KO mice and receptor antagonists to reduce the hyperalgesia response in a murine model of joint inflammation (Fernandes et al., 2011). Hypertension, particularly that induced by overstimulation of the renin-angiotensin system, possesses a large inflammatory component (Savoia and Schiffrin 2006). Therefore, we investigated pathology in our hypertensive TRPA1 WT and KO mice.

**Figure 4:**
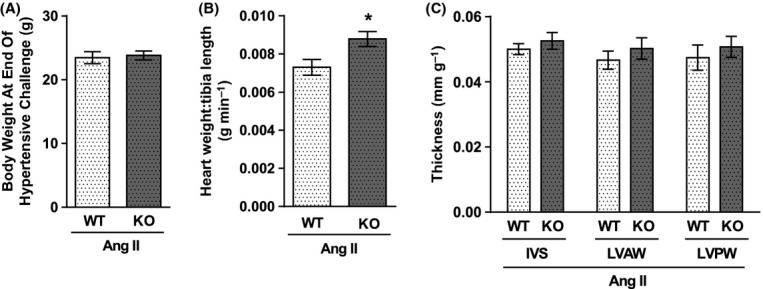
Body weight, cardiac mass, and thickness in TRPA1 WT and KO mice following 14-day infusion of angiotensin II. Body weight (A), heart weight, and tibia length were calculated at necropsy following 14 days of angiotensin II infusion (1.1 mg kg^−1^ a day) via a subcutaneously implanted osmotic minipump. Heart weight:tibia length ratio is shown in B. Thicknesses of the interventricular septum (IVS), and the left ventricle anterior (LVAW) and posterior (LVPW) walls were measured before termination using in vivo echocardiography, and are expressed as a ratio to body weight (C). *N* = 18 mice in A and B, 10–12 mice in C. Data shown ± SEM and statistics are from a two-way unpaired *t*-test, *P<0.05.

BW in TRPA1 WT and KO mice after 14 days of hypertension was similar (Fig. 4A). Heart mass-to-body mass ratios were significantly increased at day 14 compared to baseline values for both groups (Table 2). However, heart weight-to-tibia length (Fig. 4B) and LV mass-to-body mass (Table 2) ratios both showed TRPA1 KO mice to develop significantly more cardiac hypertrophy than TRPA1 WT mice. Left ventricular mass and wall thicknesses were measured using in vivo echocardiography at day 14 of angiotensin II infusion, along with functional measures. These showed further trends for increased ventricular wall thicknesses and a significantly increased left ventricular mass in hypertensive TRPA1 KO mice compared to hypertensive WT mice. In spite of these differences, cardiac function was not significantly different between genotypes (Fig. 4C, Table 2).

**Table 2:** Cardiac mass and function in TRPA1 WT and KO mice following 14-day infusion of angiotensin II

	14-day angiotensin II	
	
	TRPA1 WT	TRPA1 KO
HW:BW	5.61 ± 0.21*	6.19 ± 0.23*
(mg)	*N* = 18	*N* = 22
LV:BW	5.26 ± 0.19	5.79 ± 0.38*
(mg g^−1^)	*N* = 10	*N* = 13
(echocardiography)		
Cardiac output	14.47 ± 1.47	13.25 ± 0.92
(mL min^−1^)	*N* = 8	*N* = 10
Stroke volume	30.35 ± 2.44	26.16 ± 1.80
(*μ*L)	*N* = 7	*N* = 10
Ejection fraction	54.06 ± 2.28	60.48 ± 3.53
(%)	*N* = 8	*N* = 10
Fractional shortening	27.44 ± 1.45	32.07 ± 2.75
(%)	*N* = 8	*N* = 10

Heart weight (HW) and body weight (BW) were calculated at necropsy. All other measures were collected using in vivo echocardiography, including left ventricular mass (LV). *N* as indicated, data shown ± SEM.

* *P* < 0.05 compared to previously quoted basal values (Table 1) using a two-way ANOVA with Sidak’s post hoc test.

**Figure 5:**
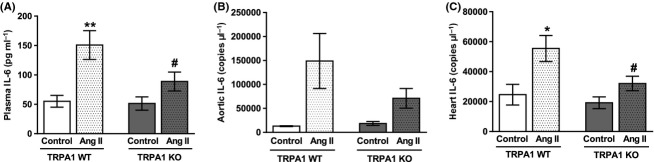
IL-6 production in TRPA1 WT and KO mice following 14-day infusion of angiotensin II or in control animals. Angiotensin II (1.1 mg kg^−1^ a day) was infused for 14 days via a subcutaneously implanted minipump. Control mice received an empty pump. At termination, blood was collected and plasma extracted. IL-6 was then measured using a multiplex enzyme-linked immunosorbent assay (ELISA) and is shown in A. *N* = 8 WT control, 16 WT angiotensin treated, 10 KO control, 17 KO angiotensin treated. mRNA was extracted from aorta and heart tissue from angiotensin II treated or control TRPA1 WT and KO mice. Following conversion to cDNA, qPCR was used to quantify IL-6 copy numbers against a standard curve. Average copy numbers are shown in B and C, respectively. *N* = 2 WT control, 8 WT angiotensin, 3 KO control, 9 KO angiotensin (B) and 9 WT control, 14 WT angiotensin treated, 10 KO control, 17 KO angiotensin treated (C). Data is presented ± SEM and statistics are from a two-way ANOVA, with Sidak’s post hoc test. *P < 0.05 or **P < 0.01 compared to respective control. #*P* < 0.05 compared to WT angiotensin II-treated animals.

IL-6 is an important cytokine in angiotensin II-induced hypertension, where the magnitude of BP increase is significantly reduced in IL-6 KO mice (Lee et al. 2006). Direct IL-6 infusion mediates cardiac fibrosis, resulting in diastolic dysfunction (Meléndez et al. 2010). IL-6 is also a “myokine,” produced by skeletal muscles during exercise and has important endocrine functions (Pedersen and Febbraio 2008). We measured plasma protein concentrations of IL-6 and messenger ribonucleic acid (mRNA) expression in the heart and aorta (Fig. 5A–C). In all cases, IL-6 was strongly upregulated during angiotensin II infusion in TRPA1 WT mice. This effect was far weaker in angiotensin II-treated TRPA1 KO mice, which showed significantly less IL-6 in plasma and mRNA expression in the heart compared to angiotensin II-treated WT mice. Similar patterns were seen in plasma IL-6 levels and aortic mRNA expression. This effect was specific to IL-6 as the plasma level of several other cytokines was not significantly different between angiotensin II-treated TRPA1 WT and KO mice (Table 3).

**Table 3:** Plasma cytokine profile in TRPA1 WT and TRPA1 KO mice following 14-day infusion of angiotensin II

	14-day angiotensin II	
	TRPA1 WT	TRPA1 KO
Plasma IL-1*β* (pg mL^−1^)	2.858 ± 1.705	2.490 ± 1.600
	*N* = 18	*N* = 17
Plasma TNF*α* (pg mL^−1^)	9.458 ± 2.835	8.821 ± 3.144
	*N* = 18	*N* = 17
Plasma IL-12 (pg mL^−1^)	273.7 ± 436.3	124.5 ± 142.4
	*N* = 18	*N* = 17
Plasma KC (pg mL^−1^)	280.8 ± 275.9	326.7 ± 323.3
	*N* = 18	*N* = 17
Plasma IFN*γ* (pg mL^−1^)	10.13 ± 9.736	6.971 ± 5.535
	*N* = 18	*N* = 17
Plasma IL-10 (pg mL^−1^)	111.3 ± 91.30	76.98 ± 53.82
	*N* = 17	*N* = 17

Angiotensin II (1.1 mg kg^−1^ a day for 14 days) was infused via a subcutaneously implanted minipump. Blood was collected at termination and plasma extracted. Cytokines were measured using a multiplex ELISA. *N* as indicated, data shown ± SEM.

### Low levels of TRPA1 mRNA expression are found in tissues of the circulatory system and brain, but predominant expression is found in DRG, where it is upregulated in hypertension

**Figure 6:**
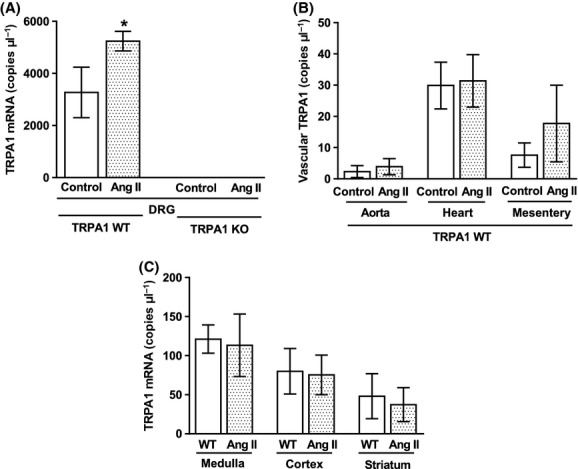
TRPA1 expression in organs involved in the control of BP and locomotion. mRNA was extracted from dorsal root ganglion neurones (DRG) (A), aorta, heart, mesenteric arterioles (B), and several discrete brain regions (C) from TRPA1 WT and KO mice following 14-day infusion with angiotensin II (1.1 mg kg^−1^ day^−1^) with a subcutaneously implanted minipump or a control minipump. Following conversion to cDNA, TRPA1 copy numbers were measured using qPCR. *N* = 3 WT control, 7 WT angiotensin treated (A and B), 2 KO control, 9 KO angiotensin treated (A) or *N* = 3 WT control, and 3 WT angiotensin treated (C). Data is presented ± SEM and statistics are from a two-way ANOVA, with all groups compared with Sidak’s post hoc test. *P < 0.05 compared to respective control.

TRPA1 gene expression is upregulated following chemical or physical nerve injury (Frederick et al. 2007). High levels of TRPA1 mRNA were found in DRG neurones from TRPA1 WT mice, with significantly elevated expression following 14 days of angiotensin II infusion (Fig. 6A). Lower copy numbers were found in tissues from several areas of the circulatory system, namely, heart, aorta, and mesenteric arterioles, where expression was not altered by angiotensin II infusion (Fig. 6B). Due to our novel findings of physical activity differences in TRPA1 WT and KO mice, we additionally investigated expression of TRPA1 in several brain areas related to locomotion control. Low levels of TRPA1 expression were found in the medulla, cortex, and striatum, with no alterations following angiotensin II infusion (Fig. 6C).

### CA-induced vasorelaxation is not dependant on TRPA1 or CGRP

**Figure 7:**
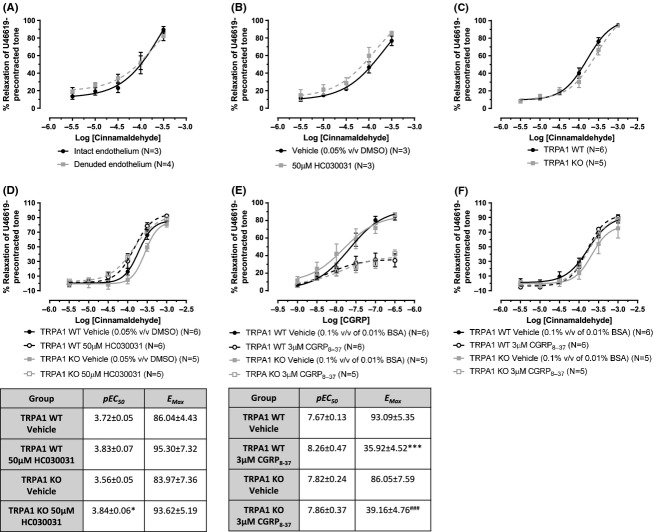
Vasorelaxation profile of the putative TRPA1 agonist cinnamaldehyde. Mesenteric arterioles from CD1 mice (A–B) or WT and TRPA1 KO mice (C–F). Vessels were preconstricted with U46619 (10 *μ*mol/L) in order to observe the vasorelaxation response. Concentration-response curves to CA were constructed in vessels (A) intact or denuded or endothelium, (B) in the presence of HC030031 (50 *μ*mo/L) or vehicle (0.05% v/v DMSO), and (C) in WT or TRPA1 KO mesenteric arterioles. (D) illustrates the effects of HC030031 (50 *μ*mol/L) or vehicle (0.05% v/v DMSO) in WT and TRPA1 KO arterioles on CA-induced relaxation, the table below contains the *pEC*50 and *E*_Max_ values. (E) shows the effects of CGRP_8-37_ (3 *μ*mol/L) or vehicle (0.1% v/v of 0.01% BSA in double deionized water) in arterioles collected from WT or TRPA1 KO mice on CGRP-induced relaxation. (F) shows the effects of CGRP_8-37_ (3 *μ*mol/L) or vehicle in arterioles collected from WT or TRPA1 KO mice on CA responses. N is indicated and data is shown ± SEM. In (D), *P < 0.05 compared to TRPA1 KO vehicle. *pEC*_50_ and *E*_Max_ from (A–C) analyzed through two-tailed unpaired *t*-test, while (D–F) analyzed by two-way ANOVA with Bonferroni’s post hoc test. In (E), ***P < 0.001 compared to TRPA1 WT vehicle, and ^###^*P* < 0.001 compared to TRPA1 KO vehicle.

CA is a well-accepted TRPA1 agonist which induces vasorelaxation that is often mediated by neuropeptide release (Louis et al. 1989; Morris et al. 1999; Hikiji et al. 2000; Grant et al. 2005; Namer et al. 2005; Graepel et al. 2011; Kunkler et al. 2011; Pozsgai et al. 2012). We have previously shown CA to cause in vivo vasodilation in the mouse paw and ex vivo vasorelaxation of mouse mesenteric arteries, both responses which were significantly reduced in TRPA1 KO mice/tissues (Pozsgai et al. 2010). Following on from our findings in this current study, showing low expression of TRPA1 in mesenteric arterioles, we have reevaluated and repeated our previous experiment investigating CA-induced relaxation of TRPA1 WT and KO mesenteric arteries, preconstricted in response to the TxA_2_ analogue U46619. Similar to our previous report, the contractile responses to U46619 were identical in TRPA1 WT and KO mesenteric arteries, both with and without functional endothelium (Data not shown). CA induced dose-dependent vasorelaxation of WT mesenteric arteries, which occurred independently of the endothelium (Fig. 7A). However, during this study we could not clearly demonstrate a TRPA1-dependent component using the TRPA1 antagonist HC030031 or vessels from TRPA1 KO mice (Fig. 7B–D).

TRPA1 is predominantly localized to sensory C fibers which release neuropeptides, including the potent vasodilator CGRP, when activated. We investigated the dependence of CA responses on CGRP. In Figure 7E, we show the CGRP peptide antagonist, CGRP_8-37_, to reduce *α*CGRP-induced vasorelaxation equally in TRPA1 WT and KO mesenteric arteries. This demonstrates the efficacy of the antagonist and also the lack of compensatory changes toward CGRP in TRPA1 KO tissues. However, CGRP_8-37_ did not alter the potency of CA-induced vasorelaxation in TRPA1 WT or KO arteries (Fig. 7F), providing evidence that the CA response is not CGRP mediated in mesenteric arterioles.

### TRPA1 KO murine mesenteric arteries from hypertensive mice show a significant defect in vasorelaxant capacity to nitric oxide

**Figure 8:**
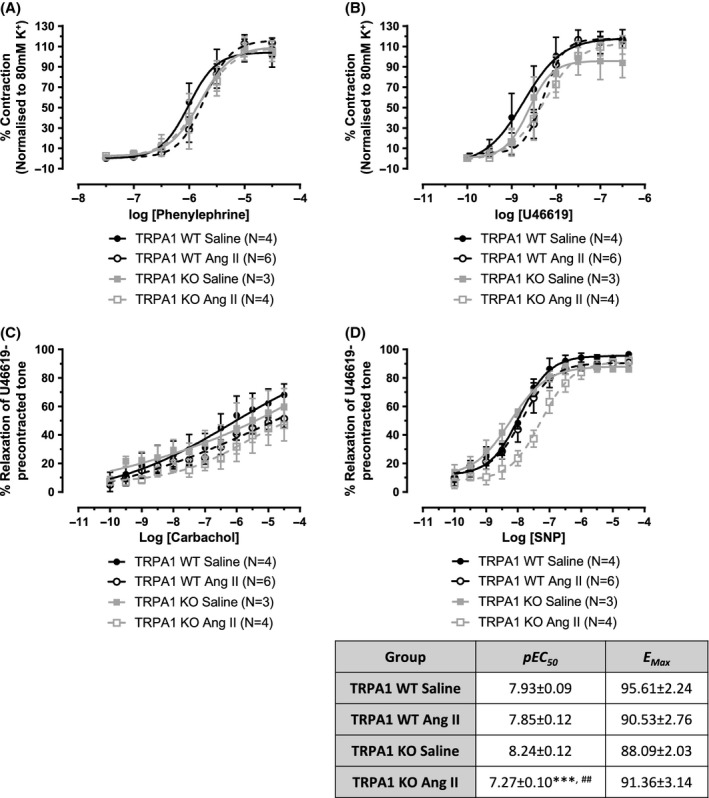
Vascular responses in arterioles from hypertensive WT and TRPA1 KO mice. Mesenteric arterioles were collected from saline-treated or Ang II-treated (1.1 mg kg^−1^ a day for 14 days) WT or TRPA1 KO mice. Concentration-response curves were constructed in response to the contractile agents phenylephrine (A) and U46619 (B). Vessels were preconstricted with U46619 (10 *μ*mol/L) to observe the relaxation response to carbachol (C) and SNP (D). *N* is as indicated and data is presented ± SEM. All statistics conducted using a two-way ANOVA with Bonferroni’s post hoc test. In (D), ***P < 0.001 is compared to TRPA1 KO saline and ^##^*P* < 0.01 compared TRPA1 WT Ang II.

Although we were unable to isolate a TRPA1- or CGRP-dependent mechanism within the CA response, there remains a possibility that TRPA1 may be expressed at functional levels in mesenteric arteries and may influence development of the vascular dysfunction associated with hypertension. In Figure 8 we show mesenteric arteries from both TRPA1 WT and KO mice demonstrated little functional deterioration during our acute model of angiotensin II-induced hypertension. No alterations in contractile capacity toward phenylephrine or U46619 were seen, alongside preserved sensitivity to carbachol-induced vasorelaxation when compared with normotensive controls (Fig. 8A–C). The only significant change was in the potency of the nitric oxide (NO) donor SNP, where a reduced response was observed in TRPA1 KO mesenteric arteries from hypertensive mice, compared to those from normotensive controls (Fig. 8D). There was no difference in NO sensitivity between vessels from normotensive TRPA1 WT and KO mice.

## Discussion

Our original hypothesis was that cardiovascular phenotype of TRPA1 KO mice would be altered, producing sensitivity to hypertension. Investigations using radiotelemetry effectively showed this was not the case, but unmasked some surprising findings. A summary of our novel results is as follows; (1) TRPA1 WT and KO mice displayed indistinguishable cardiovascular hemodynamics, cardiac morphology, and function under baseline conditions. (2) TRPA1 KO mice showed hyperactivity traits. (3) Characteristics of experimental hypertension were broadly similar in TRPA1 WT and KO mice, suggesting little obvious role for TRPA1 in systemic BP control in response to angiotensin II. (4) Increased hypertrophy, exacerbated physical activity, and blunted IL-6 production were seen in hypertensive TRPA1 KO mice. (5) TRPA1 receptor expression in DRG neurons was significantly upregulated during hypertension. (6) TRPA1 did not have an important role in CA-induced vasorelaxation. This is in keeping with the observation of relatively low levels of TRPA1 mRNA expression in the mesentery. These results suggest a lack of functional TRPA1 activity in this vascular bed.

In this study, we primarily investigated if TRPA1 deletion would alter cardiovascular phenotype and vulnerability to hypertension induced by angiotensin II infusion. Our findings demonstrate that this is not the case, as both TRPA1 WT and KO mice developed similar levels of hypertension in this model. However, a significant and new finding from this study is of a previously undescribed increase in spontaneous activity and voluntary exercise in TRPA1 KO mice compared to TRPA1 WT mice. The mechanisms involved in the control of voluntary locomotion are currently unclear, but it is likely that a variety of neuropeptides are involved in central reward mechanisms. These have been reviewed by Jordan et al. (2008) and Garland et al. (2011). Several measures of activity were collected during this study, giving independent evaluations of hyperactivity; namely spontaneous activity, voluntary exercise distance, and running speed. In all measures, TRPA1 KO mice showed consistent preferences toward increased physical activity, with increased spontaneous activity counts and wheel running activity, which was both further and faster than that seen in WT mice. These differences were exacerbated during hypertension, where in the latter stages, hypertensive TRPA1 KO mice showed significantly higher spontaneous activity counts compared to hypertensive TRPA1 WT mice. It is known that locomotion control can be a heritable trait in mice (Swallow et al. 1998) and selective breeding of mice with a high motivation to run is used to create models of attention-deficit/hyperactivity disorder (ADHD) (Rhodes et al. 2005). Several neuropeptides known to be released downstream of TRPA1 activation in the periphery, such as substance P, have strong links to mood control when released centrally (Rotzinger et al. 2010). Locomotive control is likely to derive from the central nervous system; however, significant TRPA1 expression in the brain was not found in this study. TRPA1 expression occurs in the developing rat brain (Jo et al. 2013), and is found on astrocytes from adults, where it has a role in long-term potentiation (Shigetomi et al. 2013). These findings may explain the background level of expression we have detected in our crude brain preparations. It is likely that selective isolation of discrete brain regions could reveal precise and functionally important regions of TRPA1 expression, which may influence locomotive behavior. The precise mechanistic role of TRPA1 in locomotive control has not been identified; however, increased activity preferences in TRPA1 KO mice could relate to either a higher capacity to exercise, through physical muscular changes, or an altered mental attitude favouring exercise. Investigations of these possibilities were beyond the scope of this study but are of significant intrigue, particularly as the drosophila version of TRPA1 has recently been implicated in centrally derived motor control (Flood et al. 2013).

Hypertension is considered to be a systemic inflammatory disease, characterized by enhanced oxidative stress, circulating cytokines, and vasculature remodeling, factors which are associated with faster progression to cardiovascular events and negative disease outcomes (Collier et al. 2011; Touyz and Briones 2011). Using the angiotensin II model of hypertension, we found TRPA1 to have a limited ability to modulate hemodynamics, however, some important differences to the inflammatory profile were observed. TRPA1 KO mice presented with more substantial cardiac hypertrophy, but blunted ability to increase IL-6 production; results which are difficult to reconcile. Hypertrophy is detrimental to the outcome of hypertension, leading to further increases in BP, inflammation, and cardiac dysfunction (Hein et al. 2003). By comparison, a lower level of IL-6 is suggested to be beneficial, based on KO studies where IL-6 deletion reduces angiotensin II-induced hypertension (Lee et al. 2006) and where IL-6 is reported to be a marker of diastolic dysfunction and all-cause mortality (Fisman and Tenenbaum 2010). It is likely that angiotensin II infusion leads to an increase in systemic levels of TRPA1 agonists, including reactive oxygen species and oxidized proteins (Trevisani et al. 2007; Andersson et al. 2008). The effect of TRPA1 activation on different cell types is yet to be fully elucidated, but could include alterations in protein expression profiles or effects on proliferation. Recent studies have shown TRPA1 agonists to promote cell survival at low levels (Schaefer et al. 2013), but equally, may inhibit proliferation of melanoma cells (Oehler et al. 2012) and actually promote pro-proliferation gene expression patterns within human keratinocytes (Atoyan et al. 2009). Direct links between TRPA1 activation and cell-specific changes require further study, but could account for some of the broader changes noted in this study.

In peripheral tissues, TRPA1 mRNA was predominantly expressed on DRG neurons, in agreement with previous findings (Story et al. 2003). A low-level expression was seen in all vascular tissues investigated. A study by Hatano et al. (2012) used cultured human synoviocytes and embryonic kidney cells to show TRPA1 expression can be induced by transcription factors associated with inflammation, including nuclear factor kappa B (NF*κ*B) and hypoxia-inducible factor 1 alpha (HIF1*α*). In support of these findings, we show that TRPA1 expression is upregulated on DRG neurones from hypertensive mice. However, data from Hatano et al. (2012) linked increased TRPA1 expression with inhibition of inflammatory cytokine production, which opposes our findings of similar cytokine profiles in hypertensive mice and a blunted ability to produce IL-6 in TRPA1 KO mice. Therefore, we suggest that TRPA1 may have multiple roles in inflammation which may be situation specific and deserve further study.

Several studies have investigated TRPA1-induced vasoreactivity, frequently finding this to be neuropeptide dependent (Bodkin and Brain 2010). Undoubtedly, TRPA1 agonists can mediate increased cutaneous blood flow in a neuropeptide-dependent manner (Graepel et al. 2011). We recently reported CA-induced vasorelaxation in mesenteric arterioles to be significantly reduced in TRPA1 KO mice (Pozsgai et al. 2010). In this current study, a trend toward reduced relaxation in KO mice was observed, but this did not reach significance. Furthermore, as neither a TRPA1 or CGRP antagonist affected the CA response, we suggest that TRPA1 may not have a strong ability to modulate blood flow in the mesenteric vascular bed, and that these findings may be relevant to the lack of BP phenotype in TRPA1 KO mice. The ability and mechanism of TRPA1 to induced vasorelaxation may be tissue specific, since TRPA1 expression has been found on endothelial cells in the cerebral artery of rats, inducing hyperpolarization and vasorelaxation of the underlying smooth muscle via myoendothelial gap junctions (Earley et al. 2009). This mechanism cannot feature in the mesenteric bed, as our findings show that CA-induced vasorelaxation was endothelial independent. It remains possible that CA is not activating TRPA1 in our model, despite its established role as a TRPA1 agonist. However, we are also unable to isolate a TRPA1-dependent response in the mesenteric arteriole using other proven TRPA1 agonists, including mustard oil (Earley et al. 2009) and PF-4840154 (Ryckmans et al. 2011) (Data not shown). Therefore, our current evidence suggests that there is a limited TRPA1-dependent vasoreactive response in murine mesenteric arterioles.

We proceeded to investigate the impact of hypertension on the vasoactive function of mouse mesenteric arterioles. Several studies have suggested that prolonged hypertension is associated with the development of endothelial dysfunction (Konishi and Su 1983; Taddei et al. 1995; Davel et al. 2011) and that this may be an independent risk factor for the susceptibility to cardiovascular events (Schächinger et al. 2000). Here, few changes in the contractile or relaxant ability of mesenteric arterioles from hypertensive mice were seen compared to normotensive controls. Relaxation in response to carbachol, an acetylcholine mimetic, remained unchanged and suggests the presence of healthy endothelium. A reduced potency of the NO donor SNP was observed in vessels from hypertensive TRPA1 KO mice compared to vessels from normotensive TRPA1 KO mice, but the relevance of this is not currently known. It is possible that signaling changes, reactive oxygen species, and vascular hypertrophy may have roles in this finding. Alongside our results showing increased TRPA1 mRNA expression in DRG from hypertensive mice, these findings indicate a more prominent role for TRPA1 in the control of BP and inflammation could develop in scenarios with prolonged hypertension.

## Conclusions

Conscious and unrestrained TRPA1 WT and KO mice displayed similar basal hemodynamics, cardiac morphology, and function. Comparable hypertensive responses to angiotensin II infusion were noted in both genotypes, but hypertensive TRPA1 KO mice showed blunted production of IL-6 and increased cardiac hypertrophy compared to WT mice. Both IL-6 production and cardiac hypertrophy have notable links to the prevalence of cardiovascular events and morbidity. We found little ability for TRPA1 to modulate vascular tone in arterioles from the mesenteric bed under basal or hypertensive conditions. Most strikingly, our results reveal a novel role for TRPA1 in controlling physical activity in vivo, where TRPA1 KO mice showed a preference for increased locomotion and physical exercise, which was exacerbated during hypertension. These findings emphasize the importance of studies investigating the interplay between inflammatory disease and physical activity.

## Abbreviations

ADHD: attention-deficit/hyperactivity disorder
ANOVA: analysis of variance
BP: blood pressure
BW: body weight
CA: cinnamaldehyde
cDNA: complementary DNA
CGRP: calcitonin gene-related peptide
CO: cardiac output mL/min
DMSO: dimethyl sulfoxide
DRG: dorsal root ganglion
EF: ejection fraction %
ELISA: enzyme-linked immunosorbent assay
FS: fractional shortening %
GAPDH: glyceraldehyde-3-phosphate dehydrogenase
HIF1*α*: hypoxia-inducible factor 1 alpha
IFN*γ*: interferon gamma
IL: interleukin
IVS: interventricular septum at diastole
KC: mouse KC/CXCL1
KO: knockout
LVAW: left ventricular anterior wall at diastole
LV: left ventricle
LVPW: left ventricular posterior wall at diastole
mRNA: messenger ribonucleic acid
NfκB: nuclear factor kappa B
NO: nitric oxide
PCR: polymerase chain reaction
PLA_2_G12A: phospholipase A_2_
qPCR: quantitative polymerase chain reaction
RNA: ribonucleic acid
RTPCR: reverse transcription polymerase chain reaction
SEM: standard error of the mean
SNP: sodium nitroprusside
SV: stroke volume
TNF*α*: tumor necrosis factor alpha
TRPA1: transient receptor potential ankyrin 1
TxA_2_: thromboxane A_2_
WT: wild type
